# Comparative Analysis of Minimally Invasive Therapeutic Strategies for Post-Surgical Pelvic and Retroperitoneal Lymphoceles

**DOI:** 10.3390/jcm15041346

**Published:** 2026-02-09

**Authors:** Eser Bulut, Ali Küpeli, Hasan Rıza Aydın, İsmail Taşkent, İbrahim Sibal, Neslihan Merd, Maksude Esra Kadıoğlu

**Affiliations:** 1 Department of Radiology, Trabzon Kanuni Education and Training Hospital, 61000 Trabzon, Turkey; eserbulutmd@gmail.com (E.B.); dr.ali_3383@hotmail.com (A.K.); maxudekad@hotmail.com (M.E.K.); 2 Department of Urology, Trabzon Kanuni Education and Training Hospital, 61000 Trabzon, Turkey; hrizaaydin@gmail.com (H.R.A.); ibrhmsbl@windowslive.com (İ.S.); 3 Department of Radiology, Faculty of Medicine, Kastamonu University, 37100 Kastamonu, Turkey; itaskent@kastamonu.edu.tr

**Keywords:** pelvic lymphocele, sclerotherapy, intranodal lymphangiography, lymphatic embolization, minimally invasive treatment

## Abstract

**Background/Objective:** Pelvic and retroperitoneal lymphoceles remain a clinically significant complication following pelvic surgery. The optimal minimally invasive management strategy continues to be a matter of debate. The objective of this study is to compare daily catheter drainage and catheter length of stay after percutaneous catheterization in patients with iatrogenic pelvic lymphocele who undergo sclerotherapy alone versus sclerotherapy with intranodal lymphangiography and lymphatic embolization (INL–LE). **Methods:** A total of 47 patients (55 lymphoceles) who developed symptomatic pelvic or retroperitoneal lymphoceles after oncologic pelvic surgery were retrospectively reviewed between September 2020 and April 2023. They were divided into two groups, one treated with sclerotherapy alone (24 lesions) and the other with sclerotherapy combined with INL–LE (31 lesions). The duration of catheter placement, daily drainage volume during sclerotherapy, lymphocele volume, and catheter dwell time subsequent to lymphatic embolization were compared between the two groups. **Results:** Of the 55 lymphoceles, 31 were treated with sclerotherapy plus lymphangiography/embolization (INL–LE group), whereas 24 lymphoceles were treated with sclerotherapy alone. Baseline characteristics were not different between the groups. Although initial drainage was higher in the INL–LE group, third-day drainage volume, the number of sclerotherapy sessions, and catheter dwell time were all significantly lower compared with the sclerotherapy group (all *p* < 0.001). Lesion size positively correlated with drainage volume and catheter duration, whereas embolization negatively correlated with drainage volume, the number of sessions, and catheter duration. Based on multivariate analysis, the addition of INL–LE was independently associated with a significantly shorter catheter dwell time (β = −0.803, *p* = 0.001). **Conclusions:** In this retrospective cohort, the addition of lymphatic embolization to sclerotherapy was associated with reduced drainage persistence and a shorter catheter dwell time compared with sclerotherapy alone.

## 1. Introduction

A lymphocele is a surgical complication that occurs secondary to lymphatic leakage from afferent lymphatic vessels, leading to the formation of fluid collections. It most commonly develops following lymph node dissection in oncological surgeries [1,2]. The incidence of postoperative lymphatic leakage varies widely in the literature, ranging from 9% to 61%, and approximately 2.5% to 10% of affected patients require treatment [3]. The main indications for the treatment of lymphoceles are typically superinfection or symptoms caused by the mass effect of the lymphocele compressing surrounding anatomical structures. Percutaneous catheter drainage is the first-line treatment for symptomatic lymphoceles [4,5]. However, additional interventions are often needed to reduce the drainage volume and the catheter indwelling time [4,6].

Sclerotherapy induces local inflammation in the lymphocele wall, leading to subsequent fibrosis and thereby obliterating the lymphatic leak [4]. Although sclerotherapy may be successful after a single session in some cases, the average number of sclerotherapy sessions reported in the literature ranges from one to four, with some cases requiring up to 14 sessions [6]. The need for multiple sessions arises because sclerotherapy affects only the distal leakage point, rather than the entire segment of the leaking lymphatic duct [7]. Intranodal lymphangiography (INL) can be used to investigate the presence of leaks, and if identified, lymphatic embolization (LE) can be performed under fluoroscopic guidance using a mixture of n-butyl cyanoacrylate (NBCA) and Lipiodol. The effectiveness and safety of this technique have been documented in the literature [8,9]. Recent studies have reported that lymphatic embolization performed under intranodal lymphangiography guidance may be effective in controlling postoperative lymphatic leaks. In studies directly comparing sclerotherapy and lymphatic embolization, lymphatic embolization has been shown to achieve faster clinical improvement than sclerotherapy [10]. However, despite these developments, studies evaluating the combined use of sclerotherapy and lymphatic embolization as a treatment strategy and investigating its effectiveness remain limited in the literature. A multivariate-adjusted analysis aimed at identifying predictors of catheterization duration would therefore provide a meaningful contribution to the existing body of evidence.

The objective of this study was to compare the clinical effectiveness of sclerotherapy alone versus sclerotherapy combined with lymphatic embolization in postoperative pelvic and retroperitoneal lymphoceles, focusing on drainage reduction and catheter dwell time.

## 2. Materials and Methods

This retrospective study received ethical approval from the Ethics Committee of the University of Health Sciences, Trabzon Faculty of Medicine (Approval No: 2024/155) and was conducted in accordance with the principles of the Declaration of Helsinki.

### 2.1. Study Design and Patient Selection

Between September 2020 and April 2023, 47 patients who developed symptomatic pelvic or retroperitoneal lymphoceles following oncological pelvic surgery were retrospectively reviewed. All procedures were performed at a single tertiary-care academic center. Inclusion criteria were symptomatic pelvic or retroperitoneal lymphoceles following oncologic pelvic surgery requiring percutaneous drainage. Exclusion criteria included asymptomatic lymphoceles, infected collections requiring alternative management, and incomplete clinical or procedural data.

In patients with bilateral involvement, each lymphocele was analyzed as an independent unit, yielding a total of 55 lesions. A lesion-level analytical approach was adopted, as drainage characteristics, cavity volume, catheter dwell time, and response to sclerotherapy or lymphatic embolization are primarily governed by lesion-specific factors rather than patient-level characteristics.

A total of 47 patients (55 lymphoceles) met the inclusion criteria and were allocated into two groups: the sclerotherapy group (24 lesions) and the INL–LE group (31 lesions). In the sclerotherapy group, which comprised 24 lesions, 3 patients had bilateral lymphoceles. Similarly, in the INL/LE group, 5 patients had bilateral lymphoceles. After adjusting for lesion counts, the sclerotherapy group consisted of 21 patients, while the INL/LE group included 26 patients. All patients initially received percutaneous drainage catheter placement and ethanol sclerotherapy as first-line treatment. No missing data existed for primary outcome variables.

**Figure 1:**
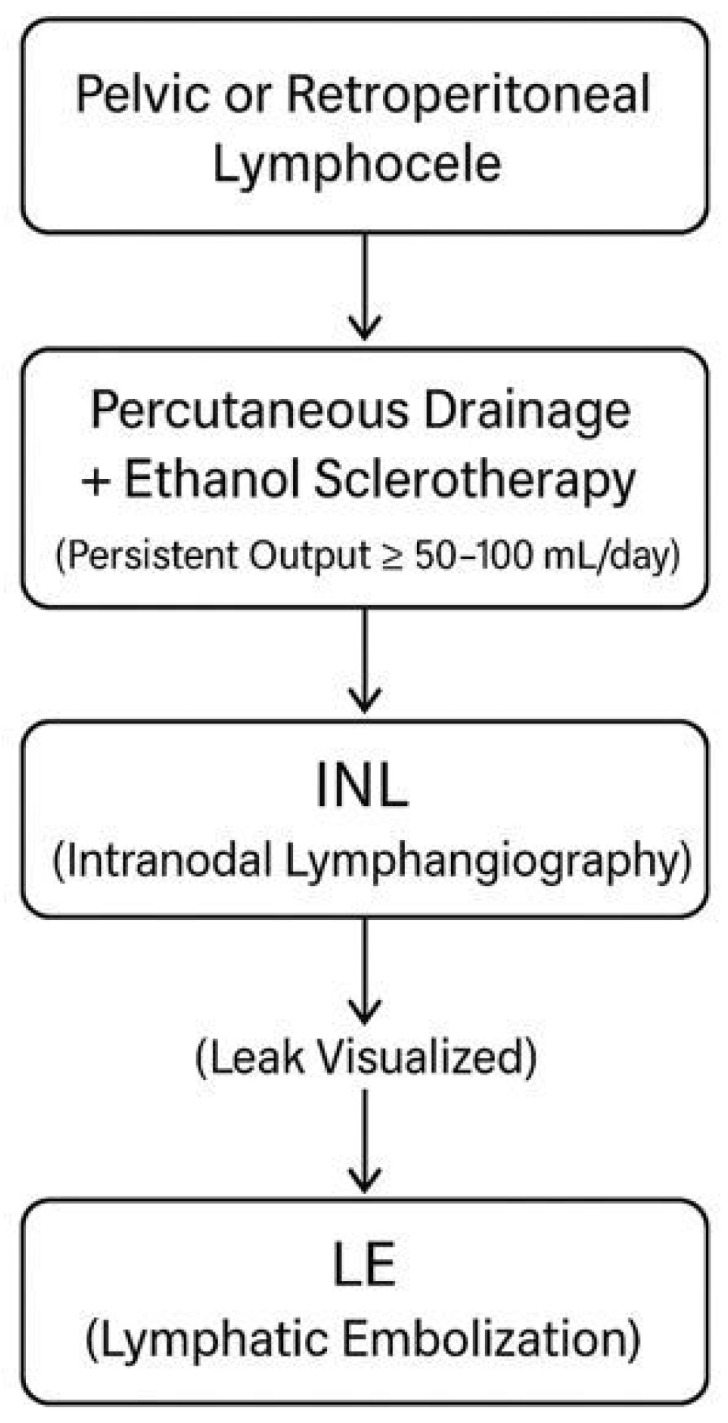
Workflow for the management of postoperative pelvic or retroperitoneal lymphoceles.

Patients were monitored daily for drainage output following sclerotherapy. On the day of catheterization, the lymphocele was evacuated and sclerotherapy was performed; this day was defined as day 0, and the subsequent day was defined as day 1. If no significant reduction in drainage volume was observed after the initial sclerotherapy (persisting at ≥50–100 mL/day) or if symptoms persisted, intranodal lymphangiography (INL) was performed on day 2. Because intranodal lymphangiography and lymphatic embolization were performed in cases with insufficient early response to sclerotherapy, treatment allocation was not randomized and may be subject to confounding by indication. In patients in whom lymphatic leakage was identified during INL, lymphatic embolization (LE) using n-butyl cyanoacrylate (NBCA) was performed during the same session or shortly thereafter. Figure 1 summarizes the treatment algorithm applied to the study population for the management of postoperative pelvic or retroperitoneal lymphoceles. Patients in whom no lymphatic leakage was detected continued with sclerotherapy alone; lymphatic leakage was not identified in only two patients, and therefore LE was not performed in these cases. During follow-up, none of the patients in the sclerotherapy-only group required additional INL/LE intervention. Likewise, no patient in the combined treatment group underwent a second INL/LE procedure.

To assess and compare the effect of the combined treatment following INL/LE performed on day 2, drainage volumes on day 3 were recorded. Accordingly, the final study cohort consisted of a sclerotherapy group (*n* = 24 lesions) and an LE group (*n* = 31 lesions). The analyzed variables included the interval between surgery and lymphocele treatment, lesion size, number of sclerotherapy sessions, drainage volumes on days 1 and 3 after intervention, and catheter dwell time.

Following the assessment of the response to the initial sclerotherapy, INL/LE was performed in order to evaluate its early impact on catheterization duration related to drainage output, as well as the associated clinical improvement. This treatment approach is routinely implemented in our clinical practice.

### 2.2. Variables

Primary outcomes included day-1 drainage volume, day-3 drainage volume, and catheter dwell time (all continuous variables). Secondary outcomes were the number of sclerotherapy sessions, leak detection on INL, and the need for escalation to lymphatic embolization. Explanatory variables included lesion size, patient age, sex, primary diagnosis, and comorbidities.

To minimize selection bias, all consecutive patients meeting the inclusion criteria during the study period were included.

As this was a retrospective cohort study, no sample size calculation was performed.

### 2.3. Sclerotherapy Technique

The procedure was performed under mild sedation. After establishing aseptic conditions, a percutaneous drainage catheter was placed under CT (CT scanner (GE Revolution EVO 128; GE Healthcare, Chicago, IL, USA)) or ultrasound (ultrasound system (RS85 Prestige; Samsung Medison, Seoul, Republic of Korea) guidance. The fluid was then completely evacuated, and its volume was recorded. Subsequently, the lymphocele cavity was filled with an equivalent volume of iodinated contrast material to assess any communication with adjacent tissues under fluoroscopic imaging. After aspiration of the contrast agent, a sclerosing agent corresponding to approximately 30–50% of the total lymphocele cavity volume was injected through the drainage catheter. The sclerosing agent was retained within the cavity for 30 min and then evacuated; thereafter, drainage was allowed to continue until 120 min after injection, after which the cavity was again emptied and the catheter was left in situ before the patient was returned to the ward. Daily drainage output was monitored, and the catheter was removed when the 24-h drainage volume decreased to less than 10 cc.

Repeat sclerotherapy sessions were performed when daily catheter output remained above the predefined threshold and failed to show sufficient reduction after the previous session. When indicated, sclerotherapy was repeated at approximately 48-h intervals, consistent with previously reported multisession protocols in the literature and aiming to reduce overall catheter dwell time [2,11].

## 3. Lymphangiography and Lymphatic Embolization Technique

Before the procedure, the inguinal area was cleaned and sterilized. Mild to moderate sedation was administered during the procedure. Under ultrasound guidance, a 25-gauge needle was advanced into the cortex-medulla junction of an inguinal lymph node. For pelvic lymphoceles, the ipsilateral lymph nodes were accessed. Once the lymph node was accessed, Lipiodol (Guerbet, Villepinte, France) was slowly injected manually under fluoroscopic guidance. The characteristic beaded appearance of Lipiodol in the lymphatic channels confirmed intralymphatic positioning. Lymphatic leakage was identified as extravasation of Lipiodol into the lymphocele cavity on fluoroscopic (Siemens Artis Zee, Germany) examination. The lymph nodes between the inguinal lymph node and the leak site were evaluated. After the leaking lymphatic channel was identified and opacified, the lymph node closest to this channel was percutaneously puncture using a 22–25 gauge needle under ultrasound guidance.

**Figure 2:**
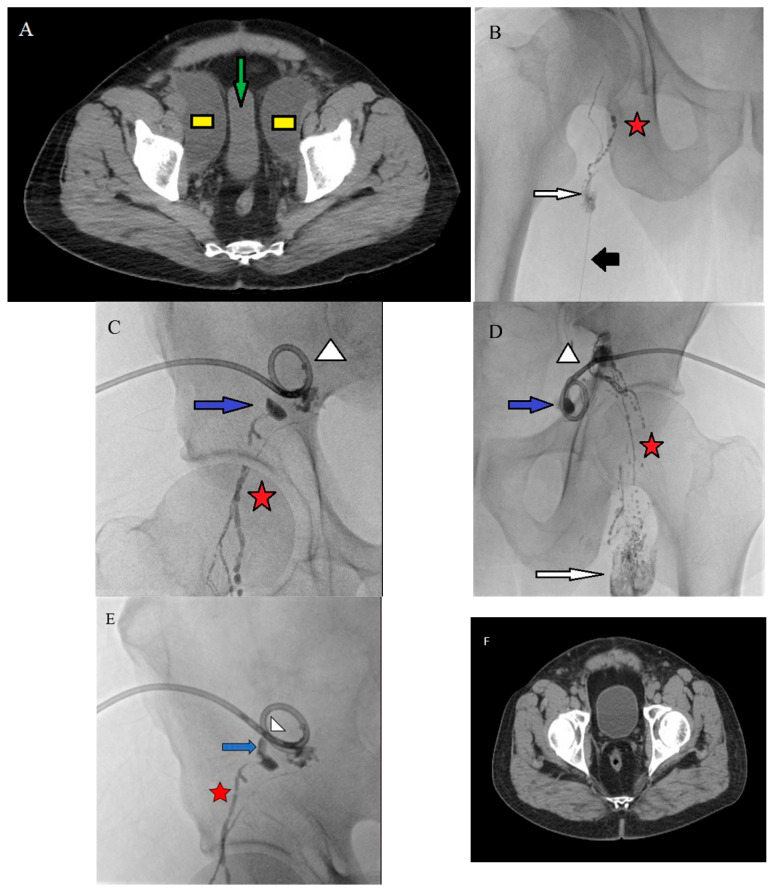
(**A**) Cystic collections consistent with bilateral pelvic lymphoceles (yellow boxes) and compression-related transverse narrowing of the urinary bladder (green arrow). (**B**) A 24G needle puncturing an inguinal lymph node (black arrow), opacification of medullary lymphatic ducts within the lymph node using Lipiodol (white arrow), and Lipiodol-opacified lymphatic channels distal to the node (red asterisk). (**C**) Drainage catheter within the right lateral pelvic collection (white arrow). Accumulated Lipiodol within the cystic cavity confirms the lesion as a lymphocele, with Lipiodol-opacified lymphatic ducts also visible (red asterisk). (**D**) Drainage catheter within the left lateral pelvic collection (white arrowhead), Lipiodol contrast material pooling within the cavity (blue arrow), Lipiodol-opacified medullary lymphatic channels of the left inguinal lymph node (white arrow), and Lipiodol-opacified lymphatic ducts distal to the node (red asterisk). (**E**) After visualization of lymphatic ductal leakage and opacification of the lymphocele cavity with Lipiodol, a Lipiodol–NBCA mixture was injected through the same lymph node access. The dense embolic material is seen to reach the injured lymphatic leak site (blue arrow) via the lymphatic duct (red asterisk) and to fill the lymphatic cavity (white arrowhead). (**F**) Post-treatment follow-up CT image demonstrating complete resolution of the lymphatic collections.

Lymphatic embolization (LE) was performed using a mixture of Lipiodol and n-butyl-2-cyanoacrylate (NBCA) (Trufill; Codman Neuro, Raynham, MA, USA). Before injecting the adhesive, 5% dextrose was injected under fluoroscopic guidance to confirm intralymphatic positioning by clearing Lipiodol from the lymphatic vessels. The ratio of NBCA to Lipiodol ranged from 1:2 to 1:10, depending on the distance between the injection site and the site of leakage, with higher dilutions used for longer distances to allow the mixture to travel further before polymerizing. Embolization was terminated when the NBCA and Lipiodol mixture was observed leaking into the lymphocele cavity. LE was not performed if no lymphatic leak was detected. Figure 2 illustrates a representative case demonstrating lymphatic leakage on intranodal lymphangiography and the subsequent lymphatic embolization procedure.

The procedural steps applied in our study are consistent with the principles described in recent clinical series utilizing intranodal lymphangiography-guided lymphatic embolization [12].

The procedures were performed by three interventional radiologists with 3–9 years of experience.

### 3.1. Procedural Variables

Technical success was defined for sclerotherapy as the successful injection of the sclerosing agent and completion of the procedure, and for lymphangiography as the successful injection of Lipiodol into the lymphatic ducts and subsequent fluoroscopic imaging. Technical success of lymphatic embolization was defined as the successful injection of NBCA into the leaking lymphatic duct or the most proximal duct close to the leak site. Clinical success was defined as the cessation of drainage from the lymphocele catheter following sclerotherapy, lymphangiography, or lymphatic embolization. After verifying the absence of catheter-related issues through appropriate catheter flushing procedures, the catheter was removed if the daily drainage volume remained at or below 10 mL for at least one day. Pre- and post-procedural changes in catheter drainage were recorded. Patients were followed at three-month intervals for possible recurrence of symptoms related to the previously drained lymphocele. The longest follow-up period was recorded as 15 months. Symptom evaluation was performed during follow-up, but no imaging was conducted solely to evaluate lymphoceles, with cross-sectional imaging reserved for the surveillance of residual or recurrent primary disease. No recurrent lymphoceles were detected during follow-up, either on clinical assessment or on cross-sectional imaging examinations.

Complications arising after the procedures were reported according to the Society of Interventional Radiology classification.

### 3.2. Statistical Analysis

All data were analyzed using the Statistical Package for the Social Sciences (SPSS 24.0 Statistical Software, SPSS Inc., Chicago, IL, USA) and the MedCalc Statistical Software version 16.8 (MedCalc Software bvba, Ostend, Belgium). Descriptive statistics, including means and ranges, were calculated for patient age, lymphocele size, drainage volume, and catheter indwelling time. The Kolmogorov–Smirnov test was used to assess data normality, and appropriate tests were selected accordingly. Accordingly, parametric tests were used for normally distributed variables, whereas non-parametric methods, including Spearman’s rank correlation analysis, were applied when normality assumptions were not met. Student’s t-test was used to compare continuous variables (daily drainage volume and duration of catheter stay before and after lymphatic embolization). Linear regression analysis was performed to investigate factors associated with catheter dwell time. Post-treatment variables, including third-day drainage volume and number of sclerotherapy sessions, were included in the regression model to reflect the clinical course and treatment burden rather than to infer causality. A *p*-value less than 0.05 was considered statistically significant.

## 4. Results

A total of 55 lesions diagnosed with pelvic or retroperitoneal lymphoceles were included in the study. Of these, 31 lesions underwent lymphangiography with or without embolization (INL–LE group), and 24 lesions were treated with sclerotherapy alone (sclerotherapy group). There were no statistically significant differences between the two groups in terms of age, sex distribution, lesion laterality, anatomical location, primary diagnosis, or comorbidities (all *p* > 0.05) (Table 1). There were no missing data for any primary outcome variables.

According to the Society of Interventional Radiology complication classification, no major procedure-related complications were observed in either treatment group. In the INL–LE group, two patients developed ipsilateral lower extremity edema following lymphatic embolization; this was classified as a minor complication and resolved with conservative medical management. In addition, pain attributable to ethanol sclerotherapy was reported in three patients in the sclerotherapy group and in two patients in the INL–LE group; this was recorded as another minor complication and resolved with medical treatment. No non-target embolization or embolic material-related adverse events were recorded.

**Figure 3:**
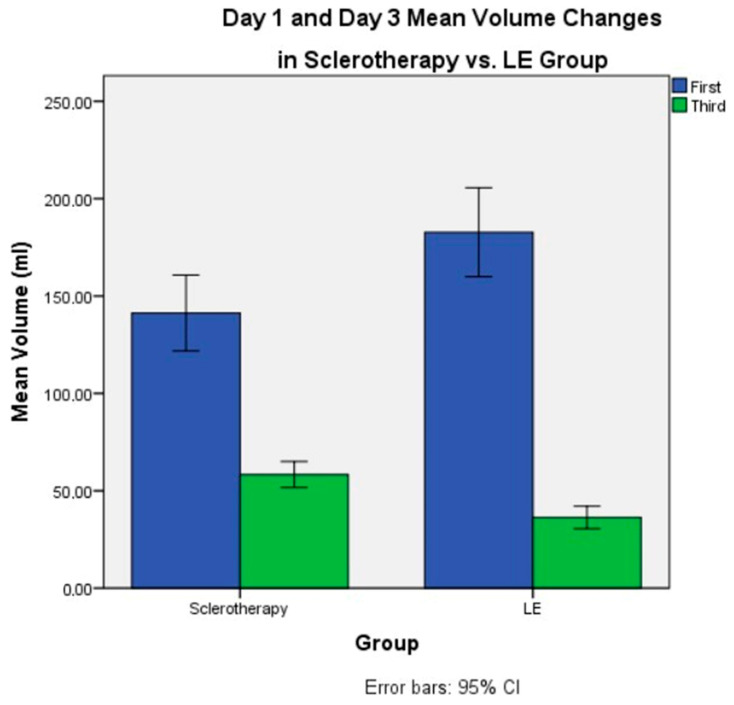
Drainage changes in groups on day one and day three.

**Figure 4:**
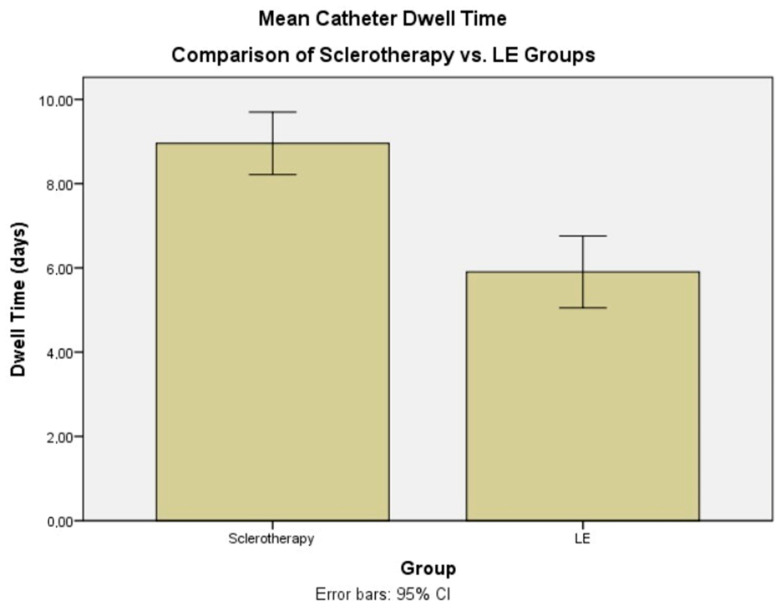
The mean catheter dwell time of the groups.

However, significant differences were observed in treatment outcomes. Despite having a higher mean drainage volume on the first day of intervention (182.7 ± 62.3 mL vs. 141.3 ± 46.2 mL, *p* = 0.010), the LE group demonstrated a significantly lower third-day drainage volume compared to the sclerotherapy group (36.3 ± 15.8 mL vs. 58.3 ± 15.8 mL, *p* < 0.001). Figure 3 schematically illustrates the changes in drainage volume in the study groups on day 1 and day 3. Furthermore, the LE group required fewer sclerotherapy sessions (2.2 ± 1.1 vs. 5.5 ± 1.3, *p* < 0.001) and exhibited a shorter mean catheter dwelling time (5.9 ± 2.3 days vs. 9.0 ± 1.8 days, *p* < 0.001) (Table 1). Figure 4 graphically summarizes and compares the mean catheter dwell time between the groups.

Spearman correlation analysis demonstrated several clinically relevant associations among lesion characteristics, procedural variables, and catheter dwell time (Table 2). Third-day drainage volume showed a strong positive correlation with both the number of sclerotherapy sessions (ρ = 0.798, *p* < 0.01) and catheter dwell time (ρ = 0.767, *p* < 0.01). In contrast, the use of INL–LE was inversely correlated with third-day drainage volume (ρ = −0.409, *p* < 0.01), sclerotherapy session count (ρ = −0.616, *p* < 0.01), and catheter dwell time (ρ = −0.454, *p* < 0.01), indicating its association with reduced treatment burden. (Table 2).

The reduction in catheter dwell time by approximately 3 days represents a clinically meaningful decrease in treatment burden.

In contrast, embolization was negatively correlated with third-day drainage (ρ = −0.409, *p* < 0.01), the number of sclerotherapy sessions (ρ = −0.616, *p* < 0.01), and catheter duration (ρ = −0.454, *p* < 0.01), indicating a potential benefit in reducing the need for repeated interventions and promoting earlier catheter removal (Table 2).

Multivariate results represent adjusted associations controlling for lesion size and initial drainage volume. To further explore predictors of catheter duration, multiple linear regression analysis was performed (Table 3). In the multivariate linear regression model, the use of INL–LE remained independently associated with reduced catheter dwell time after adjustment for lesion size and first-day drainage volume (β = −0.803, *p* = 0.001). Lesion size (β = 0.465, *p* < 0.001) and first-day drainage volume (β = 0.311, *p* < 0.001) were identified as significant positive predictors, indicating that larger lymphoceles with higher initial output tend to require longer drainage periods. In contrast, third-day fluid output (*p* = 0.603) and the number of sclerotherapy sessions (*p* = 0.608) did not reach statistical significance in the model. The use of INL–LE showed a significant independent association with reduced catheterization duration, underscoring its clinical relevance in lymphocele management (Table 3).

## 5. Discussion

In this study, patients treated with lymphatic embolization (LE) in addition to sclerotherapy showed significantly lower third-day drainage volumes and shorter catheter indwelling times compared to those treated with sclerotherapy alone. Despite having higher baseline drainage outputs, the LE group achieved faster clinical resolution, required fewer sclerotherapy sessions, and demonstrated improved overall treatment efficiency. These findings suggest that LE may be a beneficial adjunct in the management of high-output postoperative lymphoceles.

Recent advancements in interventional radiology have expanded the therapeutic options for lymphatic complications, especially following extensive pelvic or gynecologic surgeries. As highlighted by Hur et al. [13], intranodal lymphangiography and lymphatic embolization have evolved from solely diagnostic modalities into reliable therapeutic interventions for postoperative lymphatic leakage. Similarly, Majdalany et al. reported that lymphatic interventions performed under intranodal lymphangiographic guidance are rapidly advancing and becoming increasingly recognized as effective approaches for managing various postoperative lymphatic complications [14]. Their review emphasized that embolization techniques, including lymph node and lymphopseudoaneurysm embolization using NBCA, have demonstrated promising outcomes in managing chylous ascites and lymphoceles, particularly in patients unresponsive to conventional treatments. In addition, previous studies focusing on postoperative pelvic surgery have shown that increased body mass index is an independent predictor of symptomatic lymphocele formation, underscoring the contribution of patient-related factors to the clinical relevance of lymphatic complications [15]. Our study builds upon this evolving paradigm by quantitatively demonstrating, through multivariate analysis, that the combined application of sclerotherapy and embolization was associated with a significant reduction in catheter dwell time independent of lymphocele size or initial drainage volume. This finding indicates that combining sclerotherapy with embolization, rather than using sclerotherapy alone, represents not only an anatomical intervention but also a modifiable procedural variable that was associated with a favorable clinical course that can directly influence subsequent clinical outcomes.

Our findings are in line with several previous studies that evaluated the clinical benefit of LE in this setting. In the study by Moussa et al. [16] comparing LE and sclerotherapy in 46 patients, the clinical success after a single procedure was significantly higher in the LE group (83% vs. 47%, *p* = 0.011), and the catheter indwelling time was shorter (median 6 vs. 13 days). Similarly, Kim et al. [17] reported a higher three-week clinical success rate with lymph node embolization using NBCA (83.3%) compared to ethanol sclerotherapy (43.8%) in a retrospective analysis, although the difference in treatment duration and complication rates was not statistically significant.

Our study complements the work of Lee et al. [18], who investigated LE in patients with early postoperative lymphatic leaks and found that drainage volumes greater than 1500 mL/day were associated with treatment failure and prolonged catheterization (>1 week). Consistent with their observations, our correlation analyses demonstrated that larger lymphocele volumes and higher initial drainage outputs were moderately associated with longer catheter durations. Additionally, the strong positive correlation between third-day drainage and both the number of sclerotherapy sessions and catheter indwelling time suggests that early drainage dynamics may be associated with prolonged treatment and can guide timely escalation to LE.

Furthermore, Moussa et al. reported that LE alone resulted in a significant reduction in catheter output (median decrease from 210 to 20 mL/day) by the third day, with a median catheter duration of only 6 days and no need for redrainage during follow-up [8]. Similarly, Kim et al. [17] reported a higher short-term clinical success rate in the LN embolization group (83.3%) compared to sclerotherapy, despite comparable initial outputs, reinforcing LE’s role in achieving early control. In addition, Chu et al. [19] demonstrated a 100% technical and clinical success rate with a median resolution time of 7 days following LE for pelvic lymphoceles, further confirming the rapid and effective nature of this approach. These outcomes mirror our data, where LE was associated with faster control of lymphatic leakage despite a higher initial output.

Embolization’s therapeutic efficacy likely stems from its ability to directly seal the lymphatic leak at its source, in contrast to sclerotherapy, which primarily targets the lymphocele cavity. The inverse relationship between embolization and both catheter duration and sclerotherapy session count in our study reinforces this mechanism. This concept is further supported by Kim et al., who demonstrated rapid symptom resolution and reduced treatment burden following targeted NBCA glue embolization, particularly in high-output cases [17].

In the literature, most studies have compared sclerotherapy and lymphatic embolization as separate treatment modalities. In contrast, our study compares patients treated with sclerotherapy alone with those treated with a combination of sclerotherapy and lymphatic embolization, demonstrating an association with improved clinical outcomes when the combined approach was applied. The fundamental hypothesis underlying this strategy is that a synergistic effect is achieved through two distinct mechanisms: sclerosis of the lymphocele cavity with ethanol and concomitant embolization of the lymphatic duct responsible for ongoing lymphatic leakage.

In our multivariate analysis, lymphatic embolization combined with sclerotherapy remained independently associated with reduced catheter dwell time, even after adjustment for potential confounders such as lymphocele volume and first-day drainage output. Although larger fluid collections and higher baseline drainage volumes were associated with prolonged catheterization, adoption of a combined treatment strategy markedly attenuated these effects. This finding indicates that sclerotherapy combined with embolization provides more than simple anatomical sealing and can meaningfully alter the expected clinical course, particularly in high-output scenarios. Such an outcome is particularly valuable in complex cases, where timely catheter removal and reduced intervention burden are critical. By highlighting LE’s independent contribution to faster resolution, our study adds to the growing body of evidence supporting its early use in selected patients. Findings may be generalizable to similar tertiary-care centers with expertise in lymphatic interventions.

The use of intranodal access for lymphangiography and subsequent embolization in our study aligns with recent literature emphasizing its simplicity and reproducibility compared to ductal cannulation [13,20,21]. Our approach of targeting the nearest lymph node to the leaking duct, rather than cannulating the duct itself, likely contributed to our high leak detection (83.8%) and treatment success rates. By tailoring the Lipiodol-to-NBCA ratio based on leakage site proximity, we aimed to occlude the duct as close to the lymphocele cavity as possible, which may explain the significant post-procedural reduction in daily drainage and catheter duration (*p* < 0.001).

In addition, ethanol sclerotherapy, although widely used, has known limitations in high-output or large-volume lymphoceles. The reported mean catheter dwell time with ethanol sclerotherapy ranges from 17 to 22 days, with variable success. In our study, ethanol was used as the sole sclerosing agent prior to embolization. Although daily drainage volumes regressed with ethanol, a significant number of cases required escalation to LE due to inadequate resolution. This aligns with prior literature suggesting that sclerotherapy, while effective in simple cases, may be insufficient for persistent or complex lymphatic leaks [11,17]. The combined use of sclerotherapy and LE, particularly when initiated in a staged or complementary fashion, may offer synergistic benefits, as observed in our cohort.

In the literature, the complication rates associated with these procedures vary. For sclerotherapy, mild to moderate complication rates have been reported between 0% and 24% [22,23]. In our patient group, no significant complications were observed secondary to sclerotherapy. The reported complication rates for LE range from 8% to 20%. In our study, ipsilateral lower extremity edema was observed in two patients, likely due to the occlusion of the lymphatic vessels draining the affected limb during embolization. Both patients’ symptoms resolved with medical treatment.

This study has several limitations. Its retrospective, single-center design may introduce selection bias and limits control over potential confounders, which may affect the generalizability of the findings. Although 55 lymphoceles were analyzed, they originated from 47 unique patients, with some patients presenting bilateral or multi-focal lesions. As lesions were analyzed as independent units, within-patient correlation may have been introduced; however, given the limited sample size, mixed-effects modeling was not feasible, and this factor should be considered when interpreting the regression results. This analytical approach may have led to underestimation of variance and potential inflation of statistical significance. In addition, treatment allocation was based on early response to sclerotherapy, inherently assigning patients with higher-output or more complex lymphatic leaks to the INL–LE group, which may limit direct baseline comparability between treatment groups. Finally, ethanol was the sole sclerosing agent used in this study, precluding comparisons across different sclerotherapy substances.

## 6. Conclusions

In this retrospective single-center study, the addition of intranodal lymphangiography and lymphatic embolization to sclerotherapy was associated with shorter catheter dwell time and reduced treatment burden compared with sclerotherapy alone. These findings suggest that lymphatic embolization may be a useful adjunct in selected patients with persistent or high-output postoperative lymphatic leakage. Given the retrospective design and non-randomized treatment allocation, the results should be interpreted as associative rather than indicative of definitive treatment superiority.

## Figures and Tables

**Table 1:** Baseline Demographic, Clinical, and Procedural Characteristics of Patients in the Sclerotherapy and Lymphangiography/LE Groups.

Variable		Total (*n* = 55) Mean ± SD (Min–Max)	INL–LE Group (*n* = 31) Mean ± SD (Min–Max)	Sclerotherapy Group (*n* = 24) Mean ± SD (Min–Max)	*p* Value
Age		63.36 ± 8.47 (47–77)	62.84 ± 8.74 (48–77)	64.04 ± 8.26 (47–76)	>0.5
Sex	Male	36	19 (61.3%)	17 (70.8%)	0.460
	Female	19	12 (38.7%)	7 (29.2%)	
Side	Right	34	19 (61.3%)	15 (62.5%)	0.927
	Left	21	12 (38.7%)	9 (37.5%)	
Lesion size (mL)		166.47 ± 98.63 (50–492)	185.19 ± 102.68 (57–492)	142.29 ± 49.49 (50–407)	0.074
First day drainage (mL)		164.63 ± 59.12 (70–300)	182.74 ± 62.29 (70–300)	141.25 ± 46.21 (85–250)	**0.010**
Third day drainage (mL)		45.90 ± 19.12 (10–90)	36.29 ± 15.76 (10–60)	58.33 ± 15.79 (35–90)	**<0.001**
Embolic agent (mL)		2.47 ± 2.50 (0–6)	4.39 ± 1.61 (0–6)	NA	**NA**
Sclerosant count		3.67 ± 2.04 (1–8)	2.23 ± 1.12 (1–6)	5.54 ± 1.32 (4–8)	**<0.001**
Catheter length of stay		7.23 ± 2.58 (1–15)	5.9 ± 2.33 (1–12)	8.96 ± 1.76 (7–15)	**<0.001**
Diagnosis	Prostate	24 (43.64%)	11 (35.48%)	13 (54.17%)	**NA**
	Bladder	6 (10.91%)	4 (12.90%)	2 (8.33%)	
	Ovarian	8 (14.55%)	4 (12.90%)	4 (16.67%)	
	Endometrium	6 (10.91%)	4 (12.90%)	2 (8.33%)	
	Rectal	11 (20%)	8 (25.81%)	3 (12.5%)	
Comorbidity	HT	26 (47.27%)	17 (54.84%)	9 (37.5%)	**NA**
	DM	24 (43.64%)	13 (41.94%)	11 (45.83%)	
	CAD	29 (52.73%)	17 (54.84%)	12 (50%)	
Symptom	Pain	15 (27.27%)	10 (32.26%)	5 (20.83%)	**NA**
	Swelling	14 (25.45%)	7 (22.58%)	7 (29.17%)	
	Pressure	26 (47.27%)	14 (45.16%))	12 (50%)	
Location	Pelvic	39 (70.91%)	22 (70.97%)	17 (70.83%)	**NA**
	Retroperitoneal	16 (29.09%)	9 (29.03%)	7 (29.17%)	

HT = Hypertension; DM = Diabetes Mellitus; CAD = Coronary Artery Disease; LE = Lymphatic Embolization; NA = Not Applicable.

**Table 2:** Spearman Correlation Matrix of Lesion Characteristics, Procedural Variables, and Catheter Duration.

Variable	Size	First-Day Drainage	Third-Day Drainage	INL–LE	Sclerotherapy Count	Catheter Duration
Size	1	0.511 **	0.295 *	0.286 *	—	0.389 **
First-Day Drainage		1	0.375 **	0.439 **	—	0.239 *
Third-Day Drainage			1	−0.409 **	0.798 **	0.767 **
INL–LE				1	−0.616 **	−0.454 **
Sclerotherapy Count					1	0.800 **
Catheter Duration						1

ρ = Spearman’s correlation coefficient; * *p* < 0.05, ** *p* < 0.01 (1-tailed).

**Table 3:** Multiple Linear Regression Analysis for Factors Affecting Catheter Dwell Time.

Variable	Standardized Beta Coefficient (β)	*p* Value
INL–LE group	−0.803	0.001
Lesion Size	0.465	<0.001
First-Day Drainage Volume	0.311	<0.001
Third-Day Drainage Volume	0.076	0.603
Number of Sclerotherapy Sessions	0.101	0.608

β = standardized regression coefficient. Dependent variable: Catheter dwell time. Significance threshold: *p* < 0.05.

## Data Availability

The original contributions presented in this study are included in the article. Further inquiries can be directed to the corresponding author.
